# Risk of bacterial colonization by torniquet during arthroscopy of the knee joint

**DOI:** 10.3205/dgkh000502

**Published:** 2024-10-23

**Authors:** Peter Melcher, Nadine Dietze, Christoph Hellmund, Pierre Hepp, Ralf Henkelmann

**Affiliations:** 1 Department of Orthopedics and Trauma Surgery, Helios Hospital and MVZ Leisnig, Leisnig, Germany; 2 Institute of Medical Microbiology and Virology, University of Leipzig, Leipzig, Germany; 3 Department of Orthopedics, Trauma and Plastic Surgery, University of Leipzig, Leipzig, Germany

**Keywords:** Bacterial contamination, Arthroscopic knee surgery, Skin antisepsis, Surgical gloves, Elastic bandage

## Abstract

**Purpose:**

The following study investigated the risk of transmission or spread of potentially pathogenic bacteria via surgical gloves and/or with an elastic bandage to achieve a bloodless surgical site during arthroscopy.

**Methods:**

This was a single-center, prospective study performed at a level-1 trauma center. The included patients were between 18 and 65 years of age and underwent arthroscopy of the knee joint. Before arthroscopy, two skin swabs (one before and one after wrapping the leg with an elastic bandage) were taken for further microbiological analysis. In addition, the thumb and index finger of the right glove of the surgeon’s gloves and the part of the bandage covering the knee joint was kept for microbiological examination.

**Results:**

208 samples from 52 patients were included. No patient had a surgical site infection (SSI) during the follow-up period of at least 12 months. The evaluation of the microbiological findings detected contamination of the elastic wrapping material in 83% (43/52) of the cases, primarily with *Bacillus spp*. The gloves showed bacterial contamination in only two cases; a transfer to the patient’s skin was not be detected. Overall, there was no evidence of contamination from the elastic bandage or the gloves to the skin or from the skin to the wrapping material during the surgical procedure.

**Conclusion:**

Preoperative skin antisepsis is mandatory due to the risk of SSI caused by skin flora. However, in a population without a history of joint infection, the current preoperative standards for skin antisepsis seem to be sufficient to minimize SSIs during knee arthroscopy. A glove change after elastic wrapping is not necessary.

## Zusammenfassung

**Zielsetzung:**

Es sollte das Risiko der Übertragung potenziell pathogener Bakterien über Operationshandschuhe und/oder elastische Binden während einer Arthroskopie in Blutleere untersucht werden.

**Methoden:**

Es handelt sich um eine monozentrische prospektive Studie. Die eingeschlossenen Patienten waren zwischen 18 und 65 Jahre alt und unterzogen sich einer Arthroskopie am Kniegelenk. Vor der Arthroskopie wurden zwei Hautabstriche – einer vor und einer nach dem Umwickeln des Beins mit einem elastischen Verband – zur mikrobiologischen Analyse entnommen. Zusätzlich wurden der Daumen und der Indexfinger des rechten Operationshandschuhs des Chirurgen und des Teils des Verbands, der das Kniegelenk bedeckte, zur mikrobiologischen Untersuchung asserviert.

**Ergebnisse:**

Es wurden 208 Proben von 52 Patienten eingeschlossen. Kein Patient hatte während der mindestens 12monatigen Nachbeobachtungszeit eine postoperative Wundinfektion (SSI). Die Auswertung der mikrobiologischen Befunde ergab eine Kontamination des elastischen Wickelmaterials in 83% (43/52) der Fälle, hauptsächlich mit *Bacillus spp*. Die Handschuhe waren in zwei Fällen bakteriell kontaminiert; eine Übertragung auf die Haut des Patienten konnte nicht festgestellt werden. Insgesamt gab es während des chirurgischen Eingriffs keine Hinweise auf eine Kontamination vom elastischen Verband oder von den Handschuhen auf die Haut oder von der Haut auf das Wickelmaterial.

**Schlussfolgerung:**

Wegen des Risikos einer durch die Hautflora verursachten SSI ist die präoperative Hautantiseptik obligatorisch. Bei Patienten ohne Vorgeschichte von Gelenkinfektionen scheint der aktuelle Standard zur präoperativen Hautantiseptik ausreichend zu sein, um das Risiko einer SSI während einer Kniearthroskopie zu minimieren. Ein Handschuhwechsel nach elastischer Wicklung ist nicht notwendig.

## Introduction

Arthroscopic knee surgery is one of the most frequently performed orthopedic procedures. The incidence of infection after arthroscopy is very low, varying between 0.009 and 0.4% [1], [2]. However, septic arthritis is a potentially devastating postoperative complication that can include multiple revisions, significant deterioration of joint function, and even amputation [2], [3]. The analysis of possible risk factors for surgical site infections (SSI) revealed primarily patient-dependent factors, such as male gender, diabetes, obesity, and smoking [4], [5]. It also showed that long and complex surgeries as well as the use of a tourniquet have a significantly higher risk for complications including SSIs [6], [7]. To further reduce the incidence of SSIs, we investigated whether there was transmission of bacteria via surgical gloves during different arthroscopic procedures. Currently, there little evidence for this [8], [9]. Furthermore, we investigated whether there was a correlation between fluid accumulation during arthroscopy and the contamination of suture and fixation materials [10]. Current studies provide evidence that increased perioperative awareness for potential bacterial contamination followed by specific countermeasures could reduce the rate of SSIs. Therefore, the background of our study was to investigate whether contamination and possibly transmission of bacteria occurs via surgical gloves or compression material.

## Material and methods

This single-center, prospective study was performed at a level-1 trauma center. Ethical approval was given by the local ethics review committee (internal registration number 229/19-ek) in accordance with the Declaration of Helsinki and International Conference on Harmonization (Good Clinical Practice guidelines). After extensive clarification, patients provided written consent for their participation in the study. Patients between 18 and 65 years of age who underwent knee joint arthroscopy were included. Exclusion criteria were previous knee surgeries or joint infections, skin abrasions and inflammatory skin diseases such as psoriasis and neurodermatitis. To guarantee an identical procedure and sample collection, all samples were obtained by one operator. All patients were contacted 12 months after surgery and interviewed regarding SSI.

The operating room was equipped with a laminar airflow system and outwardly directed excess pressure. The surgeons wore standard sterile disposable surgical gowns and two pairs of surgical gloves. Perioperatively, all patients received antibiotic single-shot prophylaxis (Cefuroxim 1.5 g intravenously, 30 minutes before skin incision). In case of cephalosporin intolerance, 600 mg of clindamycin were administered intravenously 30 min before skin incision. All patients underwent hair removal of the surgical site with a shaver before surgery. 

The skin antisepsis was performed radially from the surgical site, moving cranially to the middle of the thigh and distally including the foot. Braunoderm^®^ (B. Braun Melsungen AG), containing the active ingredients isopropanol and povidone-iodine, was used. The skin antiseptic was applied and rubbed in multiple times using a sterile swab. The exposure time was at least 5 minutes. 

**Figure 1:**
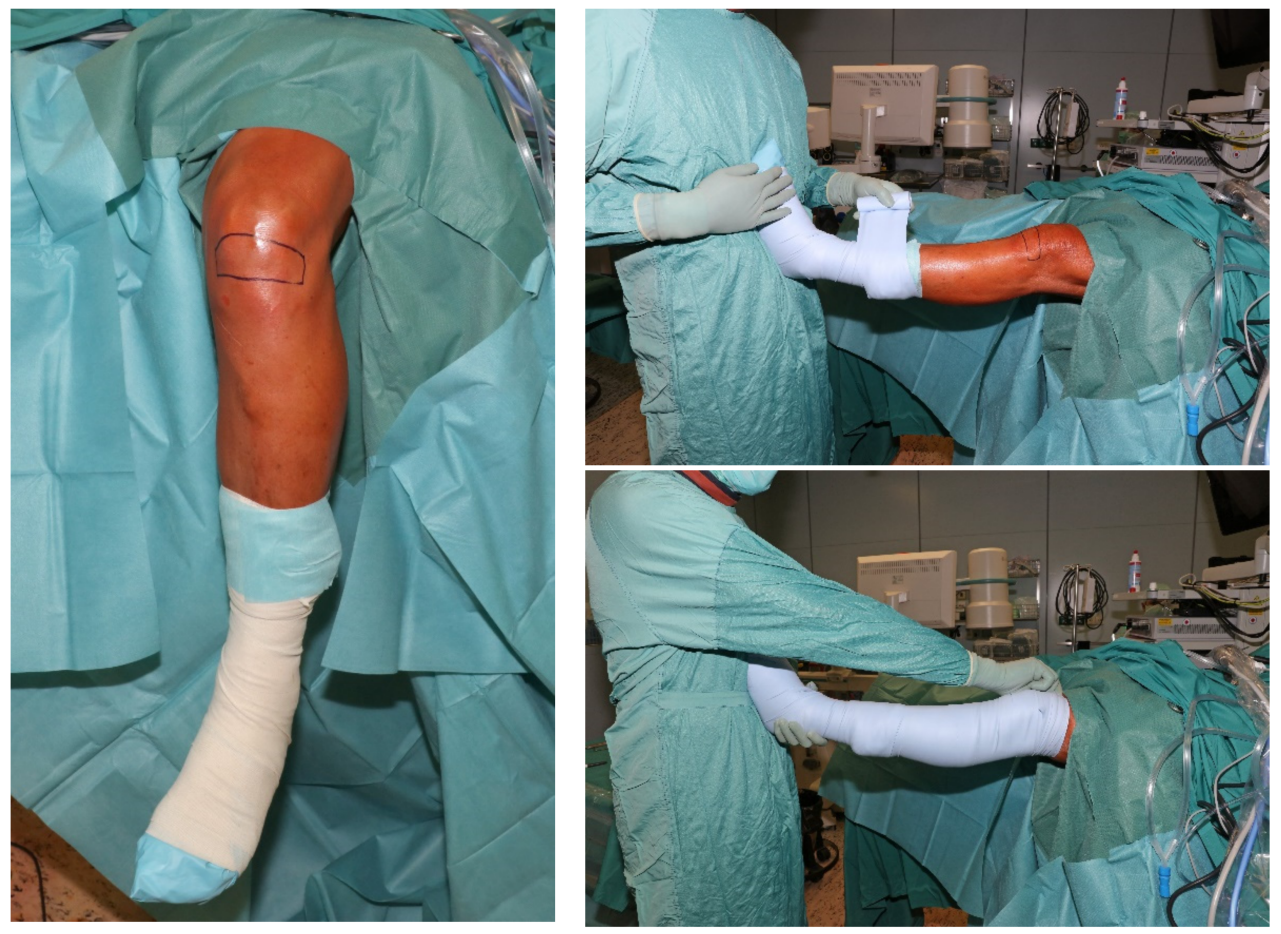
Illustration of intraoperative positioning and application of elastic bandage as well as the areas of specimen collection from the knee joint

All arthroscopies were performed in an identical manner, with the upper leg resting in a leg tray and the lower leg hanging loose (Figure 1 (Fig. 1)). After sterile draping, a swab (Copan eSwabs ^TM^, Mast Group Ltd, GB) was taken from an area of approximately 10 cm² just below the patella (Figure 1 (Fig. 1)). The leg was then wrapped with an elastic bandage to achieve a blood void (Figure 1 (Fig. 1)). The part of the bandage that came into contact with the skin above the knee joint was sampled and packaged aseptically. The tourniquet was electrically activated by switched on and a second skin swab was subsequently taken from the same area as before. Finally, the thumb and index finger of the right glove were sampled and packaged aseptically. Thus, a total of four samples per patient were collected, including two skin swabs, the thumb and index finger of the right glove and the elastic bandage. All samples were sent to the department of microbiology for further processing.

All aseptically obtained samples were examined for the presence of bacteria. Therefore, all samples were transferred to liquid (brain heart infusion) and solid culture media (blood agar) suitable for the cultivation of potentially pathogenic strains. The samples were incubated for a total of 7 days under aerobic and anaerobic conditions. In case of bacterial growth, the strains were identified by mass spectrometry (MALDI-TOF, BioMérieux, France).

## Results

A total of 208 samples from 52 patients were included in the study. No patient had a SSI during the follow-up period of at least 12 months. 

### Microbiological results

Bacterial growth was generally detected at all four sampling sites, but none of the 52 patients showed bacterial contamination at all four sample sites. Table 1 (Tab. 1) shows the results of the detected bacterial species. Out of 52 patients, only seven patients had bacterial growth at two different examination sites. In all seven patients, these were the skin swabs (before and after wrapping) and the elastic bandage. In one of the seven cases, the pathogen detected in the skin swab matched the pathogen on the elastic bandage (patient 40; Table 2 (Tab. 2)).

In 83% (43/52) of the cases, at least one colony forming unit was detected on the elastic wrap, mainly Bacillus spp. and coagulase-negative Staphylococci (CNS). In contrast, bacterial growth was detected on the gloves in only two cases, but in these cases, there was no corresponding evidence on the patient’s skin or on the elastic wrap (Table 3 (Tab. 3)).

## Discussion

Overall, there was no evidence of contamination from the elastic bandage to the skin or from the skin to the wrapping material during the surgical procedure. The detection of CNS and *Cutibacterium acnes* corresponds to the expected physiological skin flora, and only in one out of 52 cases was CNS detected on both the elastic bandage and the skin before wrapping. Therefore, contamination from the skin to the wrapping material and vice versa seems unlikely. The three cases of *Bacillus spp*. detected on the skin may be transient and should rather be considered as secondary contaminants. This shows that the preoperative antisepsis protocol seems to be sufficient for eliminating possibly pathogenic bacteria of the skin flora. These bacteria, including the weakly virulent pathogen CNS and the highly virulent *Cutibacterium acnes*, are predominantly associated with SSI knee infections [11]; however, they are more commonly found in the context of implant infections [12]. They tend not to play a role in native joint infections. Nevertheless, the fact that they could be detected on the skin before and after wrapping underlines the importance of sufficient skin antisepsis to minimize the risk of iatrogenic infections, especially since Pauzenberg et al. found that 64% of the patients tested positive for *Cutibacterium acnes* at the end of shoulder arthroscopy [13]. This may be attributed to increasingly contaminated irrigation fluid, since Bartek et al. found the contamination of irrigation fluid increased by 30% over the course of 80 minutes of arthroscopic surgery [10]. Therefore, it is concerning that there does not seem to be an effective way to reduce *Cutibacterium acnes* with standard preoperative skin antisepsis [14], [15]. When comparing an alcohol-based skin antiseptic with the addition of PVP iodine or chlorhexidine digluconate, PVP iodine showed marginal benefits concerning the aerobic flora but significantly higher efficacy against anaerobic flora of the shoulder due to the higher depth efficacy [16]. One of the most important and most frequently detected pathogens in connection with joint infections is *S. aureus* [17]. It is known to predominantly infect the synovium when introduced into the intraarticular space and may cause severe joint destruction in as little as 3 days due to its high virulence. In this study, *S. aureus* was only detected once on the wrapping material and transmission to the surgical site could not be proven.

Bacteria were most frequently detected on the elastic bandage, especially *Bacillus spp*. Only in 9 of 52 samples from the elastic bandage remained without pathogen detection. The exact moment of bacterial contamination of the wrapping material cannot be determined from this examination. and since the first skin swab showed contamination in only 6 cases, the possibility of contamination via environmental spores must be considered. Spores are ubiquitous in the environment; they are characterized by high environmental persistence and can remain on inanimate surfaces for long periods. For instance, Kobayashi et al. found that certain *Bacillus spp*. spores were able to resist 92.5°C or even higher temperatures, and grow at temperatures as low as 4°C [18]. Due to the relatively high prevalence of contamination of the elastic bandage, the possibility of primary contamination had to be ruled out. Therefore, a sample of two originally wrapped elastic bandages were incubated again for 7 days in the microbiological laboratory. Results showed no bacterial growth.

## Limitations

This study investigated the contamination of the skin, surgical gloves, and elastic bandages before arthroscopy of the knee joint of 52 patients. Patients were excluded based on age, surgical history, history of joint infection, skin abrasions and inflammatory disease. Therefore, the patients arguably most susceptible to joint infections were excluded from this study. As all surgeries were performed by the same surgeon, this study does not account for different styles of preoperative antisepsis. Also, contamination via environmental spores as well as primary contamination of the elastic wrapping material before removal from the sterile packaging cannot be ruled out. Finally, this study investigated the most common bacterial strains with regards to joint infections and does not account for every possible bacterial strain.

## Notes

### Author contributions

The authors Peter Melcher and Nadine Dietze contributed equally.

### Author’s ORCID

- Peter Melcher: 0000-0002-4747-8488

### Ethical approval 

This study was conducted after approval by the ethics committee of the University of Leipzig (internal registration number 229/19-ek). 

### Funding 

None. 

### Competing interests 

The authors declare that they have no competing interests.

## Figures and Tables

**Table 1:**
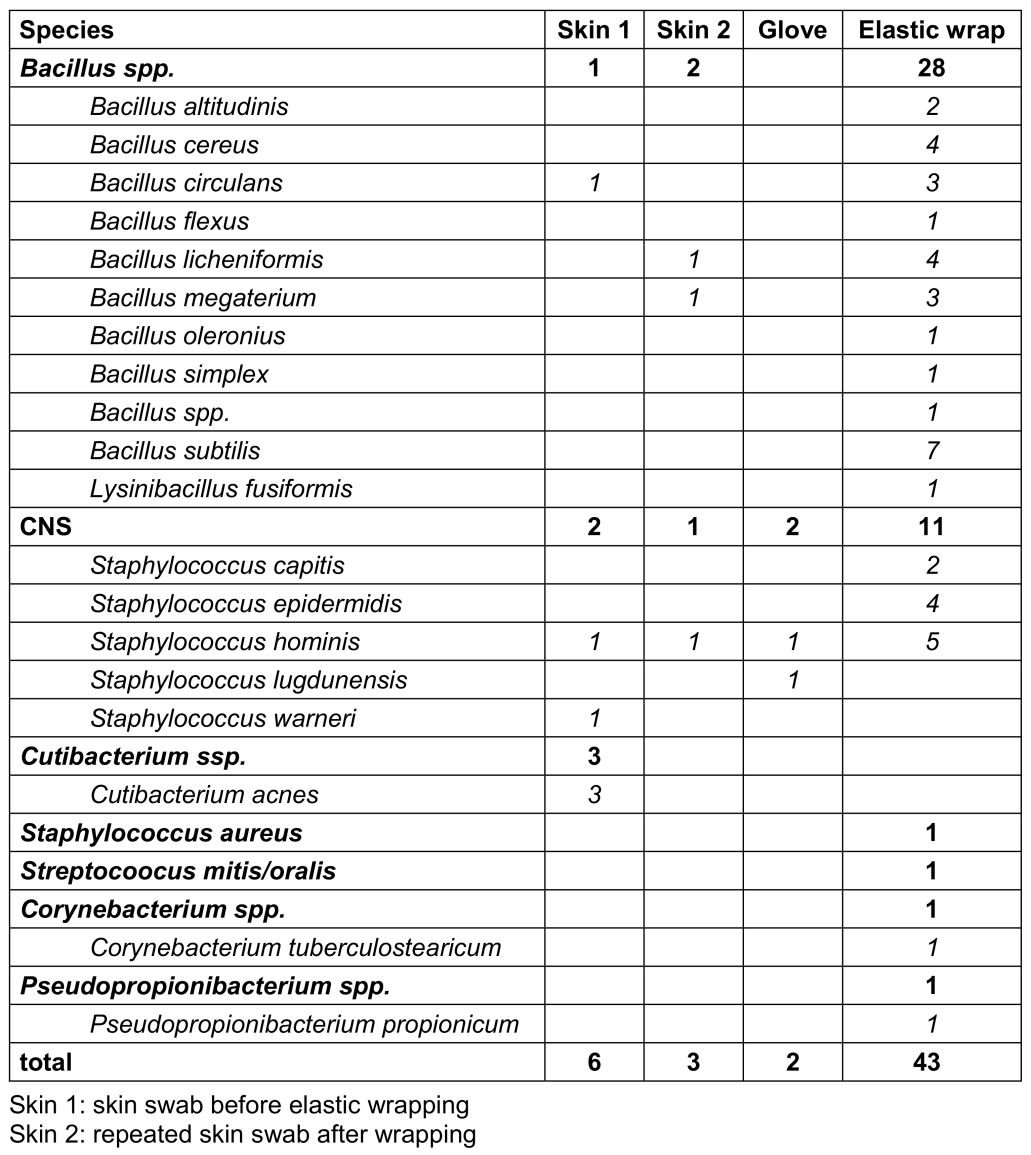
Microbiological results

**Table 2:**
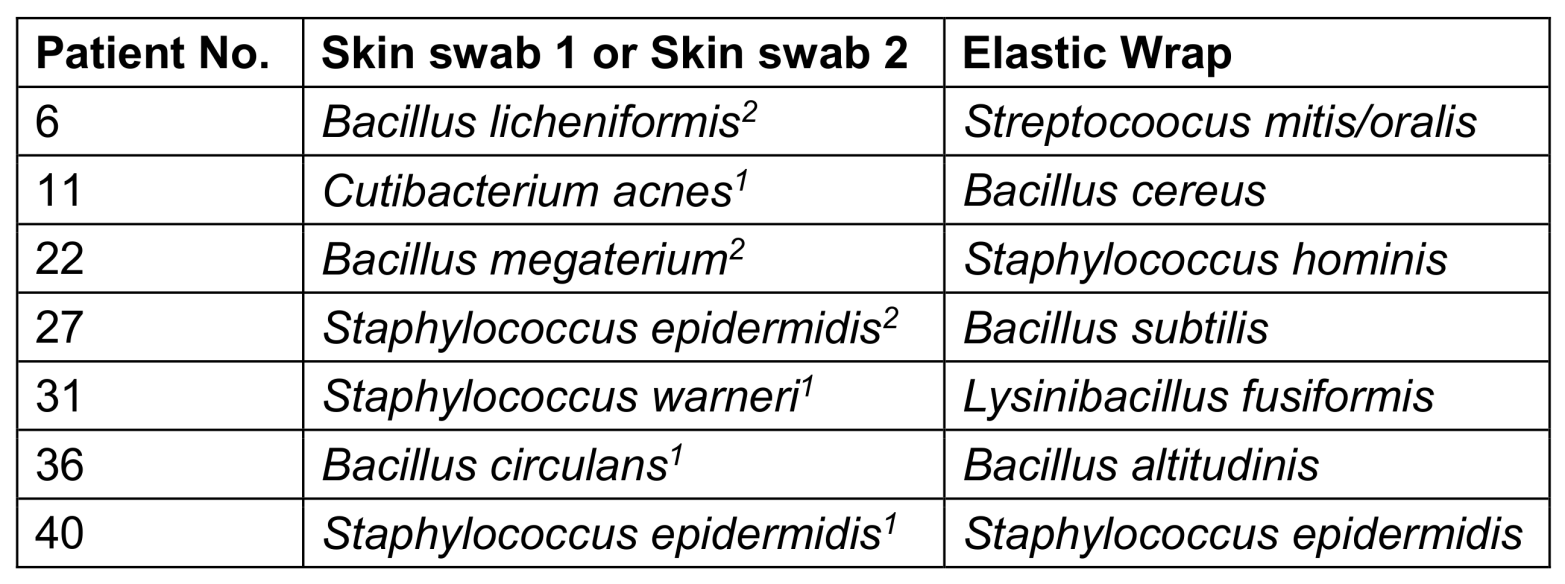
Comparison of the detected pathogens within the materials of a single patient

**Table 3:**
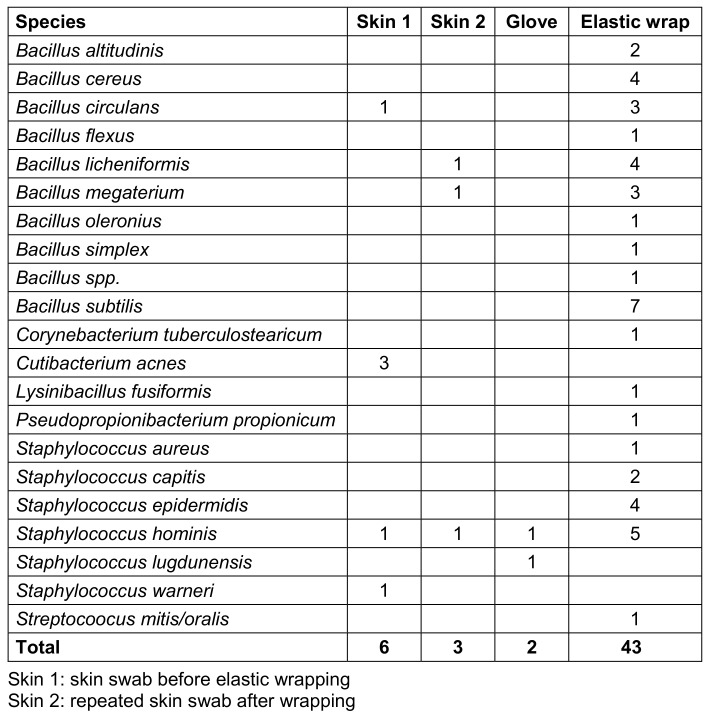
Frequency of detection of each pathogen in each sample area

## References

[1] Abram SGF, Judge A, Beard DJ, Price AJ (2018). Adverse outcomes after arthroscopic partial meniscectomy: a study of 700 000 procedures in the national Hospital Episode Statistics database for England. Lancet.

[2] Bauer T, Boisrenoult P, Jenny JY (2015). Post-arthroscopy septic arthritis: Current data and practical recommendations. Orthop Traumatol Surg Res.

[3] Balato G, Di Donato SL, Ascione T, D'Addona A, Smeraglia F, Di Vico G, Rosa D (2017). Knee Septic Arthritis after Arthroscopy: Incidence, Risk Factors, Functional Outcome, and Infection Eradication Rate. Joints.

[4] Clement RC, Haddix KP, Creighton RA, Spang JT, Tennant JN, Kamath GV (2016). Risk Factors for Infection After Knee Arthroscopy: Analysis of 595,083 Cases From 3 United States Databases. Arthroscopy.

[5] Cancienne JM, Mahon HS, Dempsey IJ, Miller MD, Werner BC (2017). Patient-related risk factors for infection following knee arthroscopy: An analysis of over 700,000 patients from two large databases. Knee.

[6] Ashraf A, Luo TD, Christophersen C, Hunter LR, Dahm DL, McIntosh AL (2014). Acute and subacute complications of pediatric and adolescent knee arthroscopy. Arthroscopy.

[7] Ahmed I, Chawla A, Underwood M, Price AJ, Metcalfe A, Hutchinson CE, Warwick J, Seers K, Parsons H, Wall PDH (2021). Time to reconsider the routine use of tourniquets in total knee arthroplasty surgery. Bone Joint J.

[8] Enz A, Klinder A, Bisping L, Lutter C, Warnke P, Tischer T, Mittelmeier W, Lenz R (2023). Knot tying in arthroplasty and arthroscopy causes lesions to surgical gloves: a potential risk of infection. Knee Surg Sports Traumatol Arthrosc.

[9] Tanner J, Parkinson H (2006). Double gloving to reduce surgical cross-infection. Cochrane Database Syst Rev.

[10] Bartek B, Winkler T, Garbe A, Schelberger T, Perka C, Jung T (2022). Bacterial contamination of irrigation fluid and suture material during ACL reconstruction and meniscus surgery : Low infection rate despite increasing contamination over surgery time. Knee Surg Sports Traumatol Arthrosc.

[11] Erice A, Neira MI, Vargas-Prada S, Chiaraviglio A, Gutiérrez-Guisado J, Rodríguez de Oya R (2018). Septic arthritis following arthroscopic reconstruction of cruciate ligaments of the knee: retrospective case review. Enferm Infecc Microbiol Clin (Engl Ed).

[12] Perry A, Lambert P (2011). Propionibacterium acnes: infection beyond the skin. Expert Rev Anti Infect Ther.

[13] Pauzenberger L, Heller V, Ostermann RC, Laky B, Heuberer PR, Anderl W (2019). Cutibacterium Acnes (Formerly Propionibacterium Acnes) Contamination of the Surgical Field During Shoulder Arthroscopy. Arthroscopy.

[14] Pauzenberger L, Grieb A, Hexel M, Laky B, Anderl W, Heuberer P (2017). Infections following arthroscopic rotator cuff repair: incidence, risk factors, and prophylaxis. Knee Surg Sports Traumatol Arthrosc.

[15] Saltzman MD, Nuber GW, Gryzlo SM, Marecek GS, Koh JL (2009). Efficacy of surgical preparation solutions in shoulder surgery. J Bone Joint Surg Am.

[16] Dörfel D, Maiwald M, Daeschlein G, Müller G, Hudek R, Assadian O, Kampf G, Kohlmann T, Harnoss JC, Kramer A (2021). Comparison of the antimicrobial efficacy of povidone-iodine-alcohol versus chlorhexidine-alcohol for surgical skin preparation on the aerobic and anaerobic skin flora of the shoulder region. Antimicrob Resist Infect Control.

[17] Shirtliff ME, Mader JT (2002). Acute septic arthritis. Clin Microbiol Rev.

[18] Kobayashi T, Azuma T, Yasokawa D, Yamaki S, Yamazaki K (2021). Spore Heat Resistance and Growth Ability at Refrigeration Temperatures of Bacillus spp. and Paenibacillus spp. Biocontrol Sci.

